# NK Cells Respond to Haptens by the Activation of Calcium Permeable Plasma Membrane Channels

**DOI:** 10.1371/journal.pone.0151031

**Published:** 2016-03-10

**Authors:** Camille Grandclément, Horst Pick, Horst Vogel, Werner Held

**Affiliations:** 1 Ludwig Center for Cancer Research, Department of Oncology, University of Lausanne, Epalinges, Switzerland; 2 Laboratory of Physical Chemistry of Polymers and Membranes, Ecole Polytechnique Fédérale de Lausanne (EPFL), Lausanne, Switzerland; Indiana University School of Medicine, UNITED STATES

## Abstract

Natural Killer (NK) cells mediate innate immunity to infected and transformed cells. Yet, NK cells can also mount hapten-specific recall responses thereby contributing to contact hypersensitivity (CHS). However, since NK cells lack antigen receptors that are used by the adaptive immune system to recognize haptens, it is not clear if NK cells respond directly to haptens and, if so, what mediates these responses. Here we show that among four haptens the two that are known to induce NK cell-dependent CHS trigger the rapid influx of extracellular Ca^2+^ into NK cells and lymphocyte cell lines. Thus lymphocytes can respond to haptens independent of antigen presentation and antigen receptors. We identify the Ca^2+^-permeable cation channel TRPC3 as a component of the lymphocyte response to one of these haptens. These data suggest that the response to the second hapten is based on a distinct mechanism, consistent with the capacity of NK cells to discriminate haptens. These findings raise the possibility that antigen-receptor independent activation of immune cells contributes to CHS.

## Introduction

Haptens are small molecules that can elicit an immune response only when attached to larger carrier molecules such as proteins. Haptens that can penetrate and chemically modify autologous molecules can “sensitize” skin when applied for the first time. Subsequent re-exposure to the same hapten applied to a different skin area of the animal can result in strong a strong inflammatory reaction or contact hypersensitivity (CHS). Hapten-induced CHS represents the prevalent animal model of allergic contact dermatitis (ACD), a delayed hypersensitivity reaction that is one of the most prevalent skin diseases in the world [1].

The induction of CHS depends on antigen presentation and the discriminatory capacity of antigen-receptors expressed by T cells together with the capacity of the adaptive immune system to form hapten-specific memory. Antigen-specific immune recognition is mediated by receptors that are assembled randomly using RAG-dependent somatic recombination and that are clonally expressed on T cells. This receptor system can recognize virtually any molecular structure and allows the immune system to respond to non-self structures when presented in the appropriate context. In hapten induced CHS, T cells recognize hapten modified self-proteins or peptides presented by dendritic cells [2]. In addition, recent work has demonstrated that NK cells can also mediate CHS suggesting that NK cells have the ability to both recognize haptens and to mediate hapten-specific recall responses [3–6]. However, how NK cells recognize and discriminate haptens or haptenated proteins in the absence of RAG-mediated antigen receptor assembly is not known.

We initially hypothesized that olfactory receptors (ORs) mediate hapten recognition by NK cells. While this hypothesis could not be confirmed, the experiments showed that NK cells can respond to several odorants and certain haptens by the activation of Ca^2+^ permeable plasma membrane channels, independent of antigen presentation. We identify a role for TRP (Transient Receptor Potential) channel C3 [7] for the NK cell response to one of two haptens known to induce NK cell-dependent CHS. Thus NK cells can recognize and discriminate haptens independent of RAG-dependent antigen receptors.

## Materials and Methods

### Odorants, haptens and inhibitors

Odorant molecules of highest available quality were from Firmenich S. A. (Geneva, Switzerland). Stock solutions of the tested substances were freshly prepared in dimethylsulphoxide (DMSO) and diluted in phosphate-buffered saline (PBS) to give the desired concentrations. Haptens, inhibitors and the TRP activators allyl isothiocyanate (AITC) and 1-oleoyl-2-acetyl-*sn*-glycerol (OAG) were purchased from Sigma-Aldrich (St. Gallen, Switzerland). Oxazolone (Oxa) was dissolved in acetone, 1-fluoro-2,4-dinitrobenzene (DNFB) in DMSO and dinitrobenzene sulfonic acid (DNBS) and 2,4,6 trinitrobenzene sulfonic acid (TNBS) in water.

### Animals

C57BL/6 mice (Harlan Horst, the Netherlands) and TRPA1 knock-out mice (B6;129P-*Trpa1*^*tm1Kykw*^/J (JAX lab) were housed under SPF conditions in individually ventilated cages. Following euthanasia by CO_2_ inhalation, spleens were excised and single cell suspensions were treated for flow cytometry as indicated below. These experiments were conducted based on procedures approved by the Service vétérinaire du Canton de Vaud (#1124.6) and performed in strict accordance with the recommendations in the Guide for the Care and Use of Laboratory Animals of the National Institutes of Health.

### Cells and Plasmids

Jurkat and NKL cells were cultured in RPMI 1640 medium containing 10% fetal bovine serum, 1% penicillin/streptomycin. Interleukin 2 (100 ng/mL) was added to NKL cultures. HEK293 cells were cultured in Dulbecco's Modified Eagle Medium/Nutrient Mixture F-12 (DMEM/F12, Life Technologies) containing 2% fetal bovine serum, 1% penicillin/streptomycin. The cells were stably transfected with the following plasmids: pCI-neo-hTRPA1, hTRPV1, hTRPV2, hTRPV3, mTRPC1, mTRPC3, mTRPC4, mTRPC5, mTRPC6 or mTRPC7 (kindly provided by Pr. Yasuo Mori, Kyoto University), pcDNA3.1-ORAI1 (Addgene #21638), ORAI2 (#16369), ORAI3 (#16370) and human STIM1-YFP (#19754) using calcium-phosphate transfection.

For CRISPR the following TRPC3 specific sequence (underlined) was cloned into Lenti CRISPR v2, a gift from Feng Zhang (Addgene plasmid # 52961) [8]:

CACCGGAGGTAGAAGCCATTCTGAAGTTT,

Jurkat cells were infected with Lenti virus based CRISPR vectors. Infected cells were isolated using puromycin selection (1 μg/mL) and subsequently cloned using limiting dilution. To verify mutations, total cellular RNA from Jurkat clones was isolated using RNAeasy kit (Quiagen) followed by reverse transcription using SuperScript III First-Strand Kit and random hexamers for priming (Invitrogen). The region of the TRPC3 mRNA that had been targeted by CRISPR was amplified by PCR using the following primers: hTRPC3 (5’- ACATAGAGAAGGAGTTCAAG and 5’- CTTGATAGCTATGGTCTGCT). The PCR product was then bulk sequenced.

### Ca^2+^ flux

Cells were loaded with Indo-1-AM (5 μM) (Sigma-Aldrich, St. Gallen, Switzerland) in Hank’s Balanced Salt Solution (HBSS) without Ca^2+^ and Mg^2+^ in the presence of 1% fcs for 45 min at 37°C. Corresponding data were obtained when cells were loaded with 2 μM of indo-1-AM in HBSS with Ca^2+^ and Mg^2+^ and 1% fcs at 37°C for 30 min. Spleen cells were then washed and surface stained with anti CD3-FITC plus DX5-PECya7 for 15 min at 4°C. The cells were resuspended at a concentration of 0.5–2*10^6^ cells/mL in HBSS containing 1% Fetal Bovine Serum, were pre-heated for 5 min at 37°C and run on a UV-equipped LSR II flow cytometer (BD Bioscience). EGTA or inhibitors were added 5–10 min before stimulation. Ionomycin (1 μg/mL) was used to measure maximal Ca^2+^ entry and Phytohemagglutinin A (PHA) (50 μg/mL) (both from Sigma-Aldrich, St. Gallen, Switzerland) or OKT3 antibodies (2 μg/mL) (prepared in house) were used to stimulate Jurkat cells via the T cell receptor (TCR).

Indo-1-AM is excited at 346 nm and emits at 398 nm when bound by Ca^2+^ and at 475 nm when unbound. The ratio of Indo-1 violet (405 nm) to blue fluorescence (510nm) (Ratio Indo-1V/Indo-1B) reports changes in calcium concentration. Baseline fluorescence was measured for 1 min. The sample was briefly removed from the cytometer to add the indicated stimulus and sample acquisition was continued for 4 min. Data were analyzed by FlowJo 7.6.4 software (Tree Star) and are plotted as the mean Indo-1V/Indo-1B ratio over time. For quantifying the magnitude of the Ca^2+^ flux response we determined the Indo-1V/Indo-1B ratio at the peak of the response relative to that of the baseline. Fold inductions of >1.3 were considered as a significant response.

## Results

### NK cells can respond to odorants and haptens in the absence of antigen presentation

**Fig 1:**
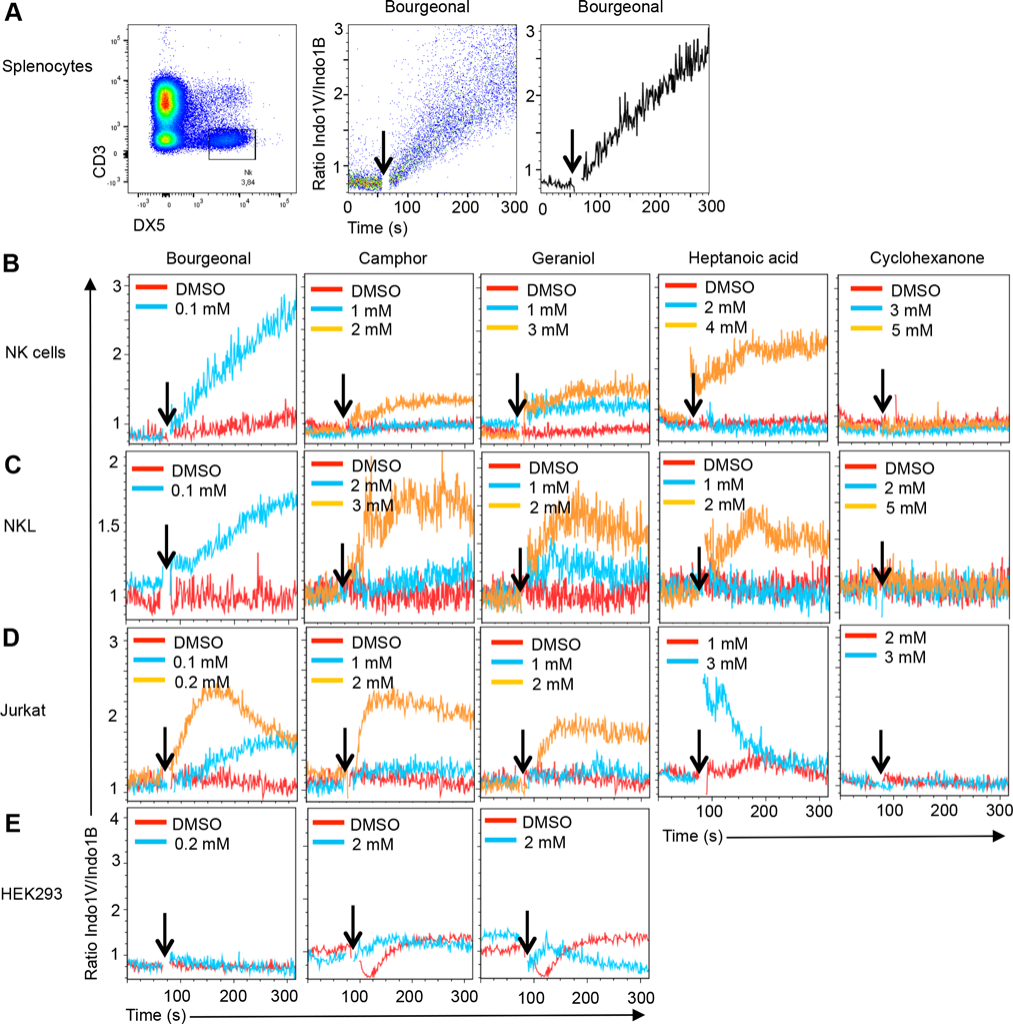
Ca^2+^ flux in gated NK cells in response to odorants. **A)** Total splenocytes from naive mice were stained with mAbs to CD3 and DX5 followed by cell loading with the Ca^2+^ sensitive dye Indo-1-AM. Gated CD3^-^ DX5^+^ NK cells were analyzed for the ratio of Indo-1 violet to blue fluorescence emission (Ratio Indo-1V/Indo-1B) over time before and after (indicated by an arrow) the addition of Bourgeonal. In the middle panel each dot represents an individual cell and the mean Indo-1V/Indo-1B ratio is plotted in the right hand panel. The indicated concentrations of several odorants, but not their solvent (DMSO), induce a Ca^2+^ flux response in NK cells from naive mice (**B**) the human NK cell line NKL (**C**) and the human T cell line Jurkat (**D**). No response is observed in the human embryonic kidney cell line HEK293 (**E**). Data are representative for 2–3 independent experiments for each condition and cell type.

We initially hypothesized that ORs, which are expressed in the olfactory epithelium where they detect volatile substances that are structurally similar to haptens **(S1 Fig)** mediate hapten recognition by NK cells. Indeed, in non-olfactory cells, like developing muscle cells or prostate cancer cells some ORs function as chemosensory receptors [9, 10]. Further, the human and mouse genomes contain large numbers of ORs (350 in human and nearly 1000 in mice) [11, 12] many of which are expressed at low levels in bone marrow NK cells (**S1 Table**). Intracellular OR signaling in olfactory neurons is mediated by G-proteins including Gα(olf) (Gnal) [13], which is expressed at very low levels in NK cells. In heterologous expression systems, OR signaling can also occur via Gq proteins, which leads to the activation of phospholipase C and the induction of an intracellular Ca^2+^ release [14]. Genes encoding Gq proteins (Gna11 and Gnaq) are expressed at considerable levels in NK cells (**S1 Table**). Collectively, this prompted us to test whether odorants induce Ca^2+^ signaling in NK cells. Consistent with the initial hypothesis several odorants, including Bourgeonal, geraniol, camphor and heptanoic acid induced Ca^2+^ signaling in primary murine NK cells, while cyclohexanone failed to do so (**Fig 1A and 1B**) (**Table 1**). Similar Ca^2+^ signaling was observed in primary T cells from naive mice (**Table 1**) and in the human NK cell and T cell lines NKL and Jurkat, respectively (**Fig 1C and 1D)** (**Table 1**). No significant response to any odorant was observed in the human embryonic kidney epithelial cell line HEK293 (**Fig 1E**). Thus lymphocytes can respond to several odorants.

**Table 1:** Odorant and hapten responses by primary lymphocytes and cell lines.

	Odorants					Hapens			
	Bour-geonal	Geraniol	Camphor	HeptanoicAcid	Cyclo-hexanone	Oxa	DNFB	DNBS	TNBS
**NK**	0.1*	1.0	2.0	4.0	-	0.4	0.25	-	-
**CD3**	0.1	1.0	1.0	4.0	-	0.4	0.25	-	-
**NKL**	0.1	1.0	2.0	2.0	-	0.4	0.25	-	-
**Jurkat**	0.1	2.0	2.0	3.0	-	0.4	0.25	2.0	1.0
**293T**	-	-	-	-	-	-	-	-	-

* lowest concentration (in mM) inducing a response as determined based on the fold change in Indo-1 emission at the peak of the response relative to the baseline. Values >1.3 were considered as a significant response. -, no response detected i.e fold change over baseline <1.3.

**Fig 2:**
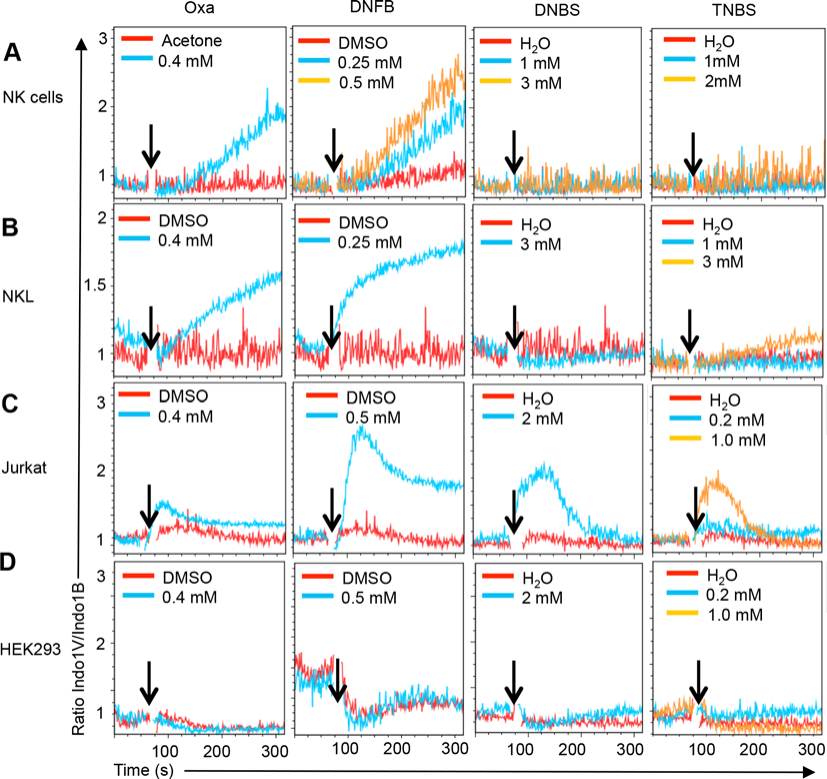
Ca^2+^ flux in NK cells in response to haptens. Ca^2+^ flux responses induced by indicated concentrations of the haptens Oxazolone (Oxa), 1-fluor-2,4 dinitrobenzene (DNFB), dinitrobenzene sulfonic acid (DNBS) and 2,4,6 trinitrobenzene sulfonic acid (TNBS) or the respective solvents (aceton, DMSO, H_2_O) in NK cells from naive mice (**A**), the human NK cell line NKL (**B**), and the human T cells line Jurkat (**C**). No response is observed in the human embryonic kidney cell line HEK293 (**D**). Data are representative of at least 2–3 independent experiments for each condition and cell type.

We next tested whether haptens induced Ca^2+^ flux in NK cells. Indeed, Oxazolone (Oxa) and 1-fluor-2,4 dinitrobenzene (DNFB) readily induced Ca^2+^ flux in primary murine NK cells and in human NKL cells (**Fig 2A and 2B**) (**S2A Fig**). In contrast, NK cells and NKL cells failed to respond to dinitrobenzene sulfonic acid (DNBS) and 2,4,6 trinitrobenzene sulfonic acid (TNBS)(**Fig 2A and 2B** and **Table 1**). Corresponding data were obtained using primary CD3^+^ T cells and human Jurkat T cells, except that Jurkat T cells also responded to DNBS and TNBS (**Fig 2C** and **Table 1)**. Under the same experimental conditions HEK293 cells did not respond to haptens (**Fig 2D**). These data reveal that haptens can stimulate lymphocytes directly and independent of antigen presenting cells, and that stimulation can occur independent of antigen receptor expression. While all four haptens can trigger Ca^2+^ flux in Jurkat cells, NK cells respond only to two of them, indicating diversity in the lymphocyte response to haptens.

### The NK cell response to odorants and haptens depends on Ca^2+^ entry

**Fig 3:**
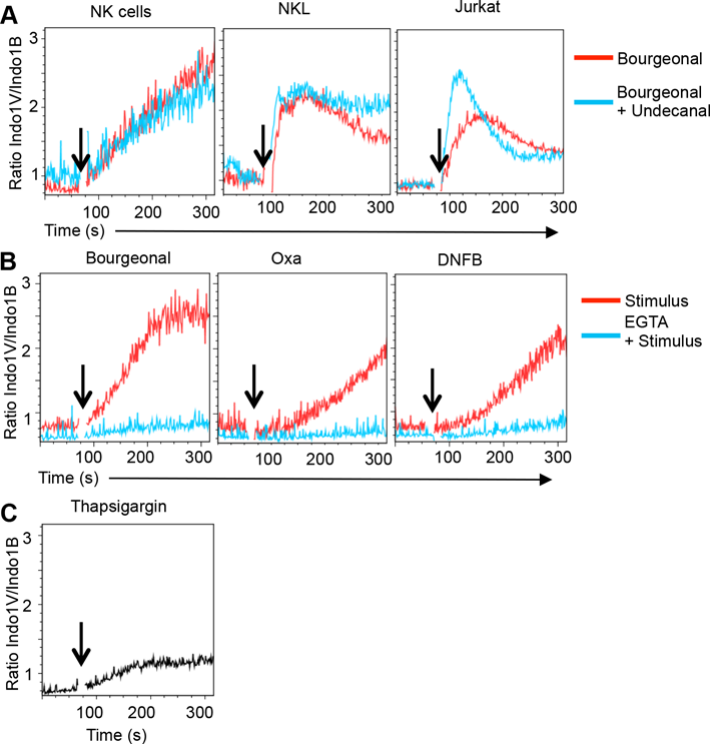
Hapten-induced Ca^2+^ flux depends on plasma membrane Ca^2+^channels. **A**) Pre-treatment with Undecanal (2.5 μM) does not inhibit Bourgeonal-induced Ca^2+^ flux in NK cells, NKL or Jurkat cells. Rather Undecanal enhanced Ca^2+^ flux. Data are representative of two independent experiments. **(B**) Chelation of extracellular Ca^2+^ using EGTA (1mM) blocks the Ca^2+^ flux response induced by Bourgeonal (0.1 mM), Oxa (0.4 mM) and DNFB (0.5 mM) in NK cells. **(C**) Ca^2+^ release from intracellular stores induced by Thapsigargin (TG) (1 μM) in NK cells when extracellular Ca^2+^ is chelated by EGTA (1mM).

We next addressed whether lymphocytes recognize odorants using OR. We initially investigated the response to the odorant Bourgeonal, which can be recognized by OR1D2 based on HEK293 transfectants [15]. Undecanal is a potent competitive antagonist of Bourgeonal-induced Ca^2+^ signaling in OR1D2 transfected 293T cells [15] as well as in the olfactory epithelium [16]. However, Undecanal failed to suppress Bourgeonal-induced Ca^2+^ flux in primary NK cells, NKL cells or Jurkat cells (**Fig 3A**). Rather, Undecanal induced Ca^2+^ flux in lymphocytes (at concentrations >2.5 μM, **S2B Fig**). Thus the lymphocyte response to Bourgeonal is in part or entirely independent of OR1D2.

More recently it was proposed that Bourgeonal and additional, structurally diverse odorants can activate Ca^2+^ channels [17]. We thus tested whether the cellular response depended on extracellular Ca^2+^. Chelation of extracellular Ca^2+^ with EGTA completely prevented the NK cell response induced by Bourgeonal and the haptens Oxa and DNFB **(Fig 3B).** Since the release of Ca^2+^ from intracellular stores (induced by Thapsigargin (TG)) was readily detectable in NK cells, even in the absence of extracellular Ca^2+^ **(Fig 3C)** we conclude that haptens do not trigger Ca^2+^ release from intracellular stores. Corresponding data were obtained in Jurkat cells (**S2C Fig**). Thus the NK cell response to haptens depends entirely on the influx of extracellular Ca^2+^, indicating a role for Ca^2+^ permeable plasma membrane channels.

### Identification of Ca^2+^ channels involved in the NK cell response to haptens

In order to identify relevant plasma membrane Ca^2+^ channels we first tested whether the Bourgeonal or hapten-induced Ca^2+^ entry occurred via CatSper channels [17]. However, two widely used inhibitors of CatSper channels (NNC55-0396 and Mibefradil) did not inhibit Ca^2+^ influx into mouse NK cells (**S3A Fig**).

**Fig 4:**
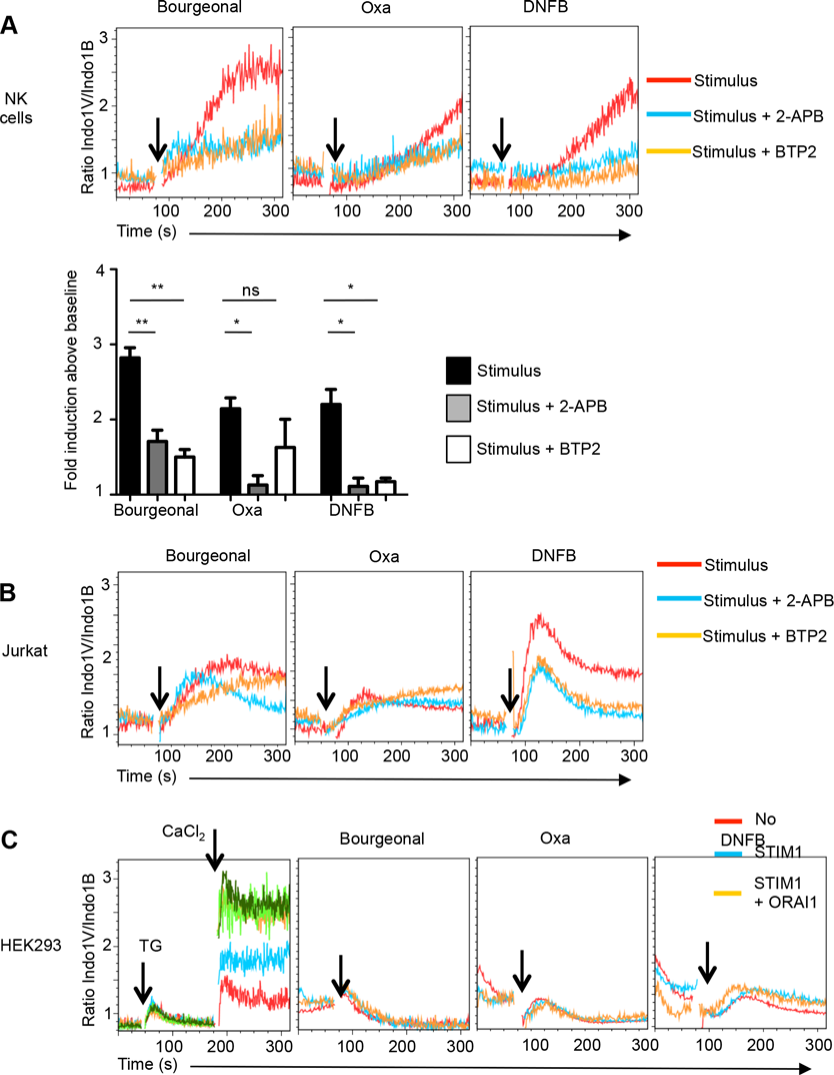
Bourgeonal-, Oxa- and DNFB-induced Ca^2+^ flux in the presence of 2-APB and BTP2. **(A**) Ca^2+^ flux induced by Bourgeonal, Oxa and DNFB in the presence of 2-APB (100 μM) and BTP2 (10 μM) in NK cells. The response by the odorant/hapten is shown in red, that in the presence of 2-APB is in blue, and that of BTP2 in yellow. The bar graph depicts the mean fold change (±SEM) in Indo-1 emission (i.e. the Indo-1V/Indo-1B ratio observed at the peak response relative to that of the baseline) induced by the stimulus alone (black bar) or the stimulus plus 2-APB (dark grey bar) or BTP2 (light grey) in NK cells from naive mice. n = 3 independent experiments, statistics as compared to stimulus alone using t-test ** p<0.01, * p<0.05, ns not significant (p>0.05). (**B**) Ca^2+^ flux induced by Bourgeonal, Oxa and DNFB in the presence of 2-APB (100 μM) and BTP2 (10 μM) in Jurkat cells (**B**). Representative of 2–3 independent experiments. **(C**) HEK293 cells were stably transfected with hSTIM1 without or with hORAI1, hORAI2 or hORAI3. Transfectants were kept in Ca^2+^-free medium and intracellular Ca^2+^ stores were depleted using Thapsigargin (TG) (1 μM). Ca^2+^ entry was measured following the addition of extracellular CaCl_2_ (1 mM). Maximal Ca^2+^ entry was detected when HEK293 cells co-expressed hSTIM1 plus hORAI1, hORAI2 or hORAI3. **(F**) Oxa (0.4 mM) and DNFB (0.25 mM) fail to induce Ca^2+^ flux in hSTIM1/hORAI transfected HEK293 cells.

The engagement of antigen receptors and certain other cell surface receptors triggers Ca^2+^ influx into lymphocytes via store operated calcium entry (SOCE) mechanisms. The release of Ca^2+^ from intracellular stores allows the calcium sensor stromal interaction molecule (STIM) 1 and 2 to activate plasma membrane CRAC (Calcium Release Activated Channel) proteins ORAI1-3 [7]. To address whether CRAC proteins are involved in odorant/hapten-induced Ca^2+^ flux, we first used the small molecule inhibitors 2-APB, which blocks ORAI1 and ORAI2, and BTP2 (YM-58483), which inhibits all three ORAI channels [18]. Both compounds prevented Bourgeonal- and DNFB-induced Ca^2+^ flux in primary NK cells **(Fig 4A)**. While 2-APB also inhibited the response to Oxa, an effect of BTP2 was not consistently observed **(Fig 4A).** Similarly, both inhibitors reduced the DNFB response in Jurkat cells, but they exerted minor or no effects on the magnitude of the Bourgeonal and Oxa response although the kinetics of these responses was altered **(Fig 4B).** While the DNFB response was consistently inhibited, NK cells and Jurkat cells may at least in part use distinct mechanisms to respond to Bourgeonal and Oxa.

To further address a possible involvement of ORAI proteins, we stably transfected HEK293 cells with human STIM1 (hSTIM1) with or without hORAI proteins. Intracellular Ca^2+^ stores were mobilized using Thapsigargin (TG) before adding CaCl_2_ to the culture medium and determining Ca^2+^ entry. Transfection of STIM1 slightly increased Ca^2+^ influx as compared to untransfected HEK293 cells. Co-transfection of STIM1 and ORAI1, ORAI2 or ORAI3 resulted in maximal Ca^2+^ entry **(Fig 4C)** [19], which demonstrates the functionality of the transfected CRAC. However, Bourgeonal, Oxa and DNFB failed to induce Ca^2+^ flux in any of these transfectants **(Fig 4C** and **S3B Fig)**. Thus ORAI proteins are not involved or not sufficient to mediate Ca^2+^ entry in response to odorants/haptens.

### A role for Transient Receptor Potential (TRP) channels for the lymphocyte response to haptens

**Fig 5:**
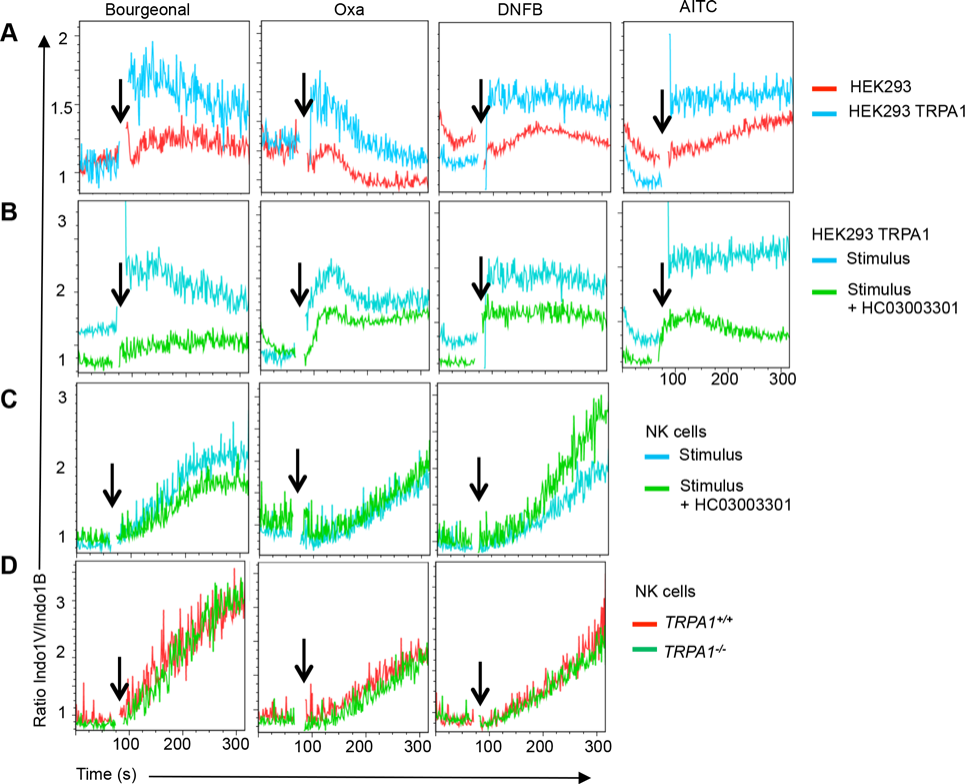
Hapten recognition by TRP channels. **(A**) Relative to untransfected control HEK293 cells (red line) stable hTRPA1 transfectants (blue line) respond to Bourgeonal, Oxa and DNFB and AITC. **(B**) The TRPA1 inhibitor HC0300301 (100 μM) inhibits Bourgeonal, Oxa and DNFB-induced Ca^2+^ flux response in HEK293 cells expressing hTRPA1 (green line). **(C**) The TRP1A inhibitor HC0300301 (100 μM) does not inhibit Oxa and DNFB-induced Ca^2+^ flux in NK cells. **(D)** Response of wild type (*TRPA1*^*+/+*^) and *TRPA1*^*-/-*^ NK cells to Bourgeonal, Oxa and DNFB. n = 4 from 2 independent experiments.

In addition to CRAC channels, Transient Receptor Potential (TRP) channels are known to contribute to Ca^2+^ influx in immune cells [7]. In addition, TRPA1 has been shown to mediate hapten responses in neurons [20]. HEK293 cells stably transfected with TRPA1 readily responded to its ligand allyl isothiocyanate (AITC) as well as to the hapten Oxa, in agreement with [21, 22]. In addition, we found that TRPA1 transfectants responded to Bourgeonal and DNFB (**Fig 5A**). All these TRPA1 responses were blocked by HC030031, a specific TRPA1 inhibitor (**Fig 5B**). However, odorant/hapten-induced Ca^2+^ flux in NK cells was not inhibited by HC030031 (**Fig 5C**). In agreement with theses findings, NK cells from TRPA1 knock out mice responded normally to Bourgeonal, Oxa and DNFB (**Fig 5D**). Thus, the NK cell response to haptens is either independent of TRPA1 or TRPA1 plays a redundant role.

**Fig 6:**
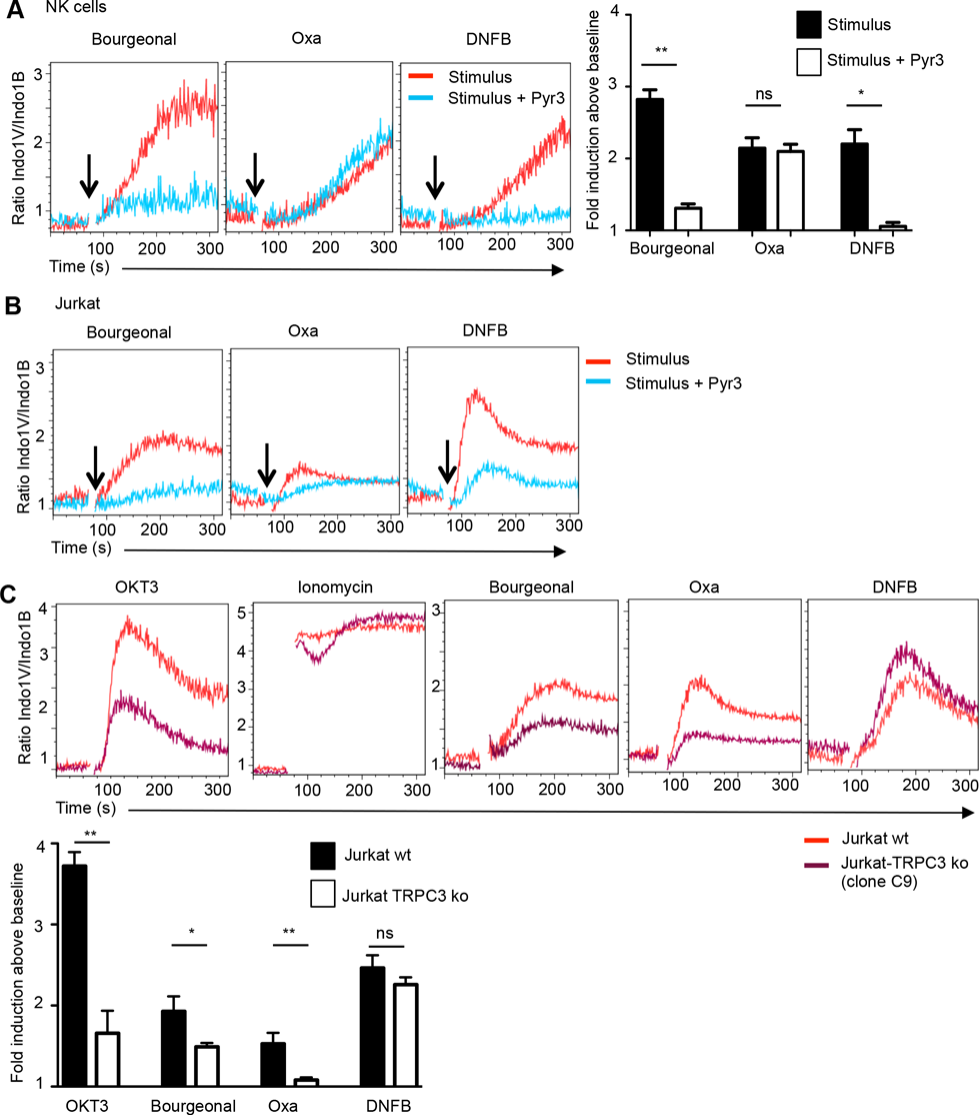
Partial TRPC3-dependence of the hapten response. (**A**) Ca^2+^ flux response by gated NK cells induced by Bourgeonal, Oxa or DNFB (red lines) in the presence of Pyr3 (10 μM) (blue lines). The bar graph depicts the mean fold change (±SEM) in Indo-1 emission (i.e. the Indo-1V/Indo-1B ratio observed at the peak response relative to that of the baseline) by the indicated compound alone (black bar) and in the presence of Pyr3 (open bar). n = 3, Statistics as compared to compound alone using t-test ** p<0.01, * p<0.05, ns not significant. (**B**) Ca^2+^ flux response by Jurkat cells induced by Bourgeonal, Oxa or DNFB (red lines) in the presence of Pyr3 (10 μM) (blue lines). **(C**) Ca^2+^ entry into *TRPC3* ko (purple line) or wild type Jurkat cells (red line) induced by OKT3 antibody (2 μg/mL), Ionomycin (1 μg/mL), Bourgeonal (100 μM), Oxa (400 μM) or DNFB (250 μM). The bar graph depicts the mean fold change (±SEM) in Indo-1 emission (i.e. the Indo-1V/Indo-1B ratio observed at the peak response relative to that of the baseline) by the indicated stimulus in TRPC3 wild type (wt) (black bar) and a TRPC3 mutant clone (open bar). Data are compiled from 3–4 independent experiments (n = 3–7). Statistical significance ** p<0.01, * p<0.05, ns not significant based on unpaired t-test (p>0.05).

The two inhibitors 2-APB and BTP2 (YM-58483), which impacted the hapten response, do not only restrain ORAI but also TRP channels in particular TRPC subfamily members (TRPC1, TRPC3, TRPC5 and TRPC6 for 2-APB ([23] and references therein) and TRPC3 and TRPC5 for BTP2: [24]). This prompted us to further test the more selective TRPC3 inhibitor Pyr3 [25]. Pyr3 readily inhibited Bourgeonal and DNFB induced Ca^2+^ influx in primary NK cells and Jurkat cells **(Fig 6A and 6B** and **S4A Fig).** Pyr3 also reduced the Oxa response in Jurkat cells, but had no effect or even increased the Oxa response in NK cells **(Fig 6A and 6B** and **S4A Fig)**. Thus TRPC3 seems to be involved in certain hapten/odorant responses in lymphocytes.

To definitely address a role for TRPC3, we generated *TRPC3* gene mutant Jurkat cells using CRISPR technology [8]. Mutations in *TRPC3* were characterized by PCR amplification and sequencing of the relevant portion of the *TRPC3* mRNA (**S4B Fig**). A *TRPC3* knock out clone (C9) displayed strongly diminished Ca^2+^ flux responses upon cross-linking the T cell receptor (TCR) using anti-OKT3 mAb (**Fig 6C**) or using Phytohemaglutinin (PHA) (**S4C Fig**), in agreement with the analysis of TRPC3 knock down cells [26], confirming that TRPC3 contributes to SOCE. On the other hand Ionomycin induced Ca^2+^ influx was not impaired (**Fig 6C**). On the other hand TRPC3 mutant cells showed significantly reduced responses to Bourgenoal and Oxa while the effect on the DNFB response was not significant (**Fig 6C**). Corresponding data were obtained with a second TRPC3 mutant Jurkat clone (E6) (**S4D Fig**). Thus the genetic approach establishes that TRPC3 is involved in the lymphocyte response to at least one hapten.

## Discussion

NK cells can mediate hapten-specific recall responses thereby contributing to contact hypersensitivity (CHS) [3, 5, 27]. However, it has not been known whether NK cells can directly respond to haptens. Here we show that two haptens that are known to induce NK cell dependent CHS (Oxa and DNFB), induce a prominent Ca^2+^ flux response. Interestingly, two other haptens, which are known to induce delayed type hypersensitivity, but have an unknown capacity to induce NK cell dependent CHS, failed to induce a Ca^2+^ flux response in NK cells, but did so in Jurkat T cells. Thus some but not all haptens can stimulate NK cells directly. These responses are thus independent of antigen receptor expression and independent of antigen presentation.

We used standard procedures to estimate Ca^2+^ flux responses in individual lymphocytes using the cell-permeable ratiometric Ca^2+^ indicator Indo 1 and flow cytometry [28, 29]. However, elevated Indo 1 concentrations can buffer intracellular calcium and thus dampen measurements of free calcium. Although this does not affect the main conclusions of the manuscript it is likely that the differences in calcium signaling are underestimated.

The cellular response induced by haptens was independent of the release of Ca^2+^ from intracellular stores and depended entirely on the influx of extracellular Ca^2+^, indicating that Oxa and DNFB activated Ca^2+^ permeable plasma membrane channels. Using a heterologous expression system we found no evidence for an involvement of ORAI proteins, which form plasma membrane Ca^2+^ channels that are activated in lymphocytes via certain cell surface receptors [7]. On the other hand we confirmed and extended findings that TRPA1 can mediate hapten responses in a heterologous expression system [21, 22]. However, even though *TRPA1* mRNA was detected, NK cell responses to haptens were not altered in the presence of a specific TRPA1 inhibitor or when NK cells lacked a functional *TRPA1* gene. Mutant Jurkat cells provided evidence that *TRPC3* is necessary for the response to the hapten Oxa and the odorant Bourgeonal. These findings identify a first component of the lymphocyte response to one hapten. They indicate that the response to a second hapten (DNFB) is based on a distinct mechanism, consistent with a capacity of lymphocytes to discriminate haptens.

Even though TRPC3 was required for the response to Bourgeonal and Oxa, TRPC3 transfected HEK293T cells failed to respond Bourgeonal and Oxa (**S5 Fig**). Of note, these TRPC3 transfectants were functional based on the response to 1-oleoyl-2-acetyl-*sn*-glycerol (OAG) (**S5 Fig**) and heterologous HEK293T were competent to respond to haptens when transfected with TRPA1 (**Fig 5A**). We conclude that TRPC3 is involved but is not sufficient to mediate the Oxa response in lymphocytes. We tested a possible role for several additional TRP family members including mTRPC1, mTRPC4, mTRPC5, mTRPC6, mTRPC7, hTRPV1, hTR6PV2 or hTRPV3 by stable transfection into HEK293 cells. However, none of these transfectants responded to Bourgeonal, Oxa or DNFB.

There are a number of possible explanations for the finding that TRPC3 is involved, but is not sufficient to mediate an Oxa response. One possible explanation is that the Oxa response depends on a complex containing TRPC3. Indeed, the closely related TRPC family members TPRC3, TPRC6 and TPRC7 can form heteromeric complexes [30]. Thus the Oxa response may depend on a heteromeric TRPC complex that includes TRPC3. This may account for the somewhat contradictory effects of the inhibitors. Inhibition of TRPC3 may not suffice to block the function of the putative complex, while the absence of TRPC3 may prevent the formation of the complex. Finally, the composition of the putative complex may vary between Jurkat cells and NK cells and this may alter the effects of the inhibitors.

In addition to heteromeric TRPC complexes, there is evidence that members of the TRPC family interact with STIM [31]. Indeed, TRPC3 was initially considered as a candidate CRAC gene. However following the identification of ORAI, TRPC channels were no longer thought to be part of the CRAC channel or to be activated by Ca^2+^ store depletion. Notwithstanding, published findings [32, 33] and our data (**Fig 6C**) show that the absence of TRPC3 reduces antigen receptor induced Ca^2+^ entry in Jurkat T cells, indicating an accessory role of TRPC3 in SOCE. It was thus possible that the Oxa response similarly depended on a cooperation of TRPC3 with STIM. However, preliminary experiments indicate that HEK293 cells co-expressing TRPC3 and STIM1 do also not respond to haptens.

Finally, TRPC3 can be activated by diacyl glycerol (DAG) and derivatives thereof, possibly via intermediate proteins [26, 34] indicating that TRPC3 can be activated indirectly based on the production of metabolites. It is thus possible that haptens indirectly activate TRPC3 in lymphocytes using specific signaling pathways. Thus, despite the identification of TRPC3 as a first component of the lymphocyte response to haptens the precise basis for the Oxa response in lymphocytes remains to be defined. In addition the possible diversity of mechanisms mediating hapten responses in lymphocytes remain to be addressed.

In conclusion, we show that NK cells and T cells can respond to haptens independent of antigen presentation and antigen receptor specificity. Thus T cells can respond in two distinct ways: Hapten recognition via the TCR based on antigen presentation or the activation of Ca^2+^ channels independent of the specificity of the TCR. It will thus be important to determine to what extent the activation of Ca^2+^ channels in lymphocytes contributes to the induction of CHS.

## Supporting Information

S1 Fig Structures of haptens and some odorants used in this study. (TIF)

S2 Fig The response to odorants and haptens depends on Ca2+ entry. A) Effect of hapten titration on Ca2+ entry. Total splenocytes were exposed to the indicated concentrations of Bourgeonal, Oxa or DNFB and the Ca2+ flux response of gated NK cells was measured over time. B) Undecanal induces Ca2+ flux in NK cells at concentrations >2.5 μM. C) Bourgeonal (0.1 mM) or Oxa (0.5 mM) fail to induce Ca2+ flux in Jurkat cells when extracellular Ca2+ is chelated by EGTA (2 mM). Thapsigargin (TG) (1 μM) induced Ca2+ release from intracellular stores is detected in Jurkat cells when extracellular Ca2+ is chelated by EGTA (2 mM).
(TIF)

S3 Fig Identification of Ca2+ channels involved in the NK cell response to haptens. A) CatSper inhibitors do not block hapten-induced Ca2+ entry into NK cells. The CatSper inhibitors NNC55-0396 (1 μM) and Mibefradil (5 μM) do not to reduce Bourgeonal, Oxa or DNFB-induced Ca2+ entry into primary NK cells. Rather these compounds may enhance Ca2+ entry. B) HEK293 cells were stably transfected with hSTIM1 without or with hORAI1, hORAI2 or hORAI3. Bourgeonal (100 μM) Oxa (0.4 mM) and DNFB (0.25 mM) failed to induce Ca2+ flux in any of the transfectants.
(TIF)

S4 Fig Role of TRPC3 for the hapten response. A) Ca2+ entry into gated NK cells (top) or Jurkat cells (bottom) induced by Bourgeonal (100 μM), Oxa (400 μM) or DNFB (500 μM for NK cells and 100 μM for Jurkat cells) in the presence of a low dose of Pyr3 (2 μM). B). Sequence of the targeted portion of TRPC3 available in NCBI, determined in wild type Jurkat cells and in the mutant clones C9 and E6. The TRPC3 species amplified from C9 has a 1bp insertion (+1), which leads to a premature stop and thus loss of function. E6 has a 6bp deletion, which removes 2 amino acids from the cytoplasmic portion of TRPC3. E6 is thus likely a hypomorphic rather than a null mutation. The sgRNA-targeting the TRPC3 sequence is shown in green, the protospacer-adjacent motif (PAM) sequence is in red. C) Ca2+ flux response induced by Phytohaemagglutinin (PHA) (50 μg/mL) in Jurkat cells (red line) and TRPC3 mutant Jurkat clone C9. D) Ca2+ entry into TRPC3 mutant clone E6 (blue line) and wild type Jurkat cells (red line) induced by Phytohaemagglutinin (PHA) (50 μg/mL), OKT3 antibody (2 μg/mL), Ionomycin (1 μg/mL), Bourgeonal (100 μM), Oxa (0.4 mM) or DNFB (0.25 mM).
(TIF)

S5 Fig TRPC3 transfected HEK293T cells do not respond to haptens. HEK293T were stably transfected with TRPC3 cDNA (blue line) and stimulated with Bourgeonal, Oxa or DNFB or the TRPC3 ligand 1-oleoyl-2-acetyl-sn-glycerol (OAG).
(TIF)

S1 Table Expression of genes coding for OR and G-proteins in NK cells. Analysis of bone marrow NK cells from wild type mice for the expression of OR and selected G-proteins genes in bone marrow NK cells. Shown are the 20 most highly expressed OR genes and selected G- Proteins.
(DOCX)
