# Supplementary figures and images for: NK Cells Respond to Haptens by the Activation of Calcium Permeable Plasma Membrane Channels
Source: PLoS One. 2016 Mar 10;11(3):e0151031. doi: 10.1371/journal.pone.0151031 (PMC4786276; doi:10.1371/journal.pone.0151031)

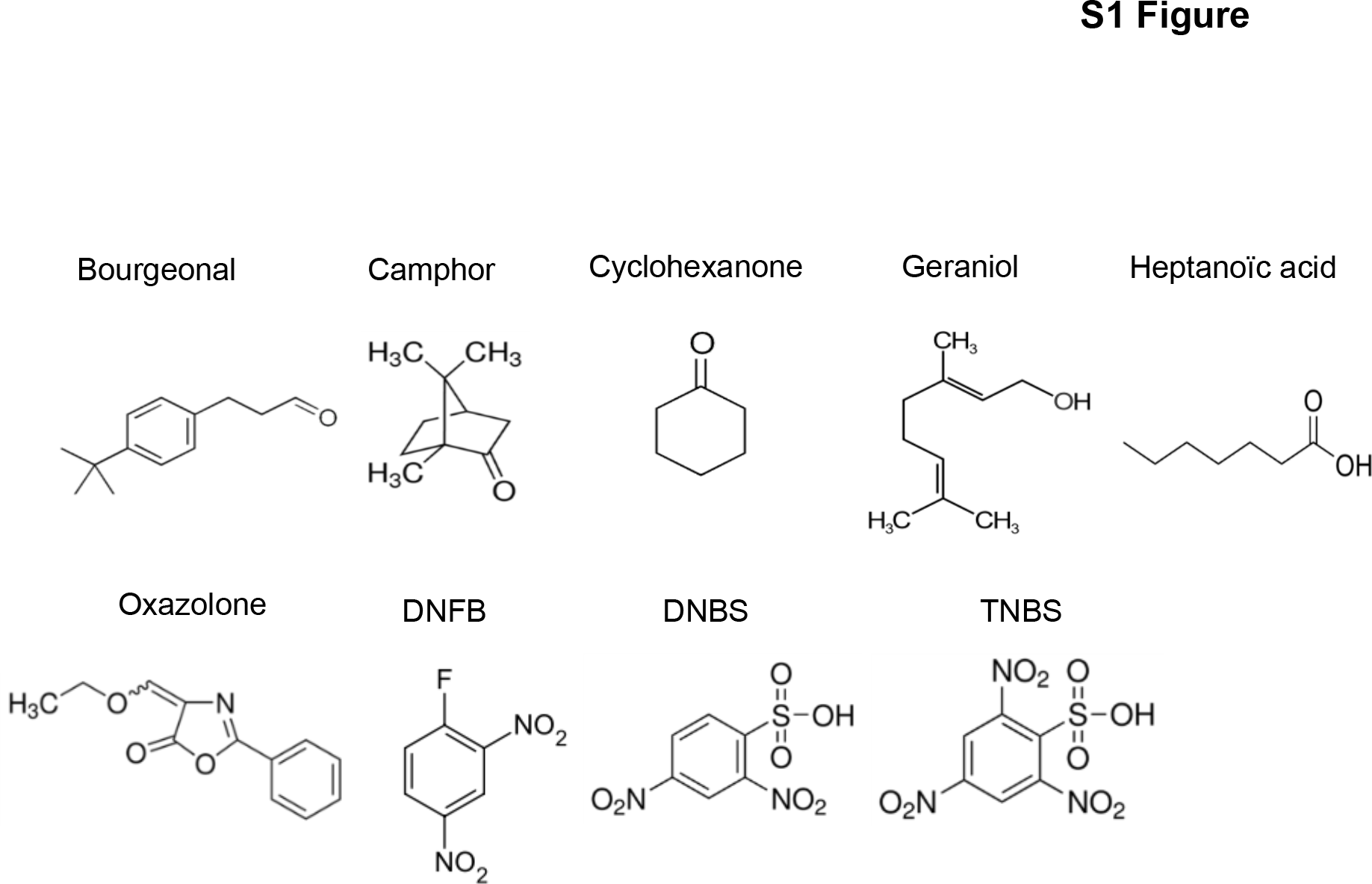

Supplement: S1 Fig — (TIF) [file pone.0151031.s001.tif]

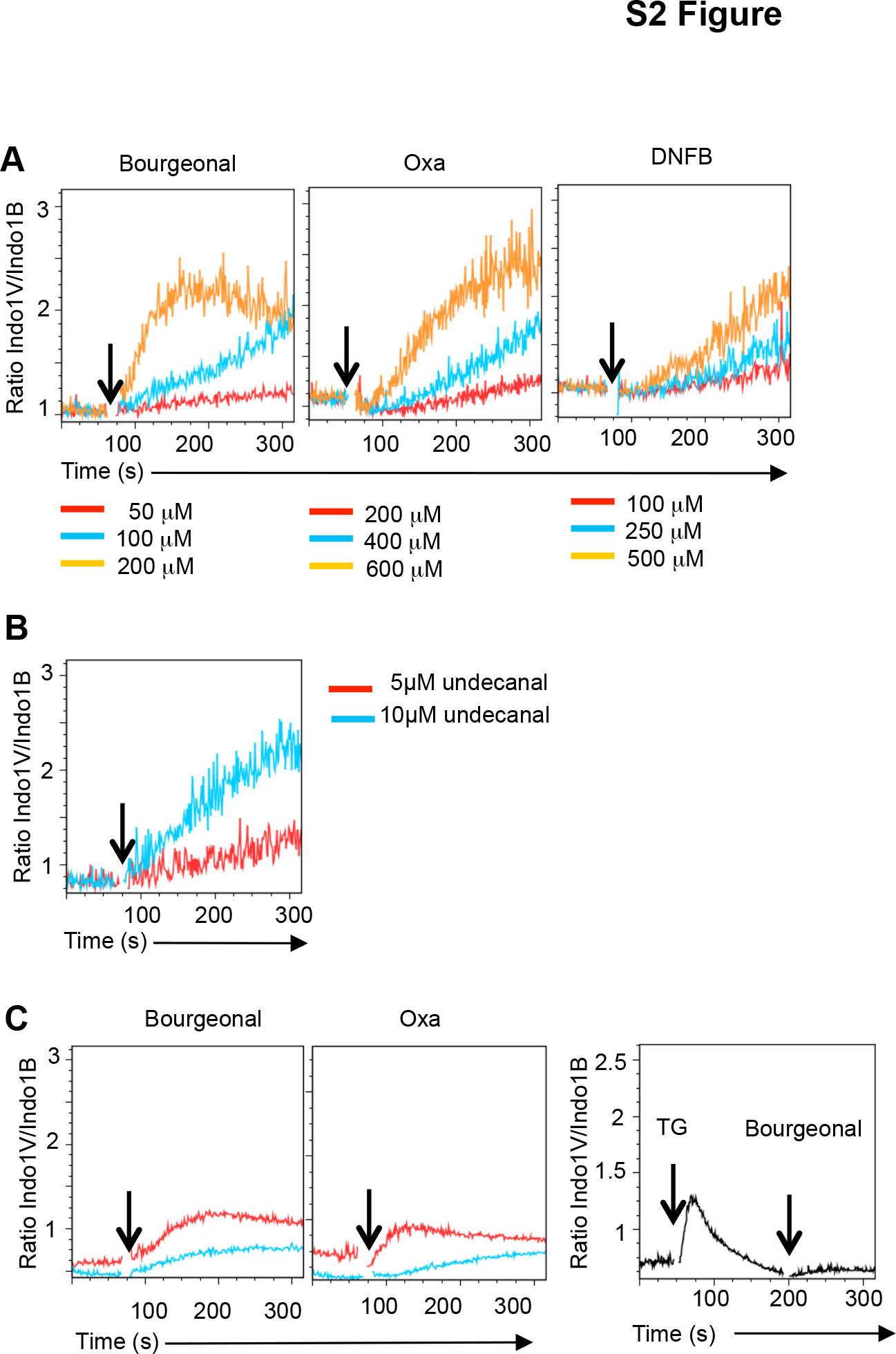

Supplement: S2 Fig — A) Effect of hapten titration on Ca2+ entry. Total splenocytes were exposed to the indicated concentrations of Bourgeonal, Oxa or DNFB and the Ca2+ flux response of gated NK cells was measured over time. B) Undecanal induces Ca2+ flux in NK cells at concentrations >2.5 μM. C) Bourgeonal (0.1 mM) or Oxa (0.5 mM) fail to induce Ca2+ flux in Jurkat cells when extracellular Ca2+ is chelated by EGTA (2 mM). Thapsigargin (TG) (1 μM) induced Ca2+ release from intracellular stores is detected in Jurkat cells when extracellular Ca2+ is chelated by EGTA (2 mM). (TIF) [file pone.0151031.s002.tif]

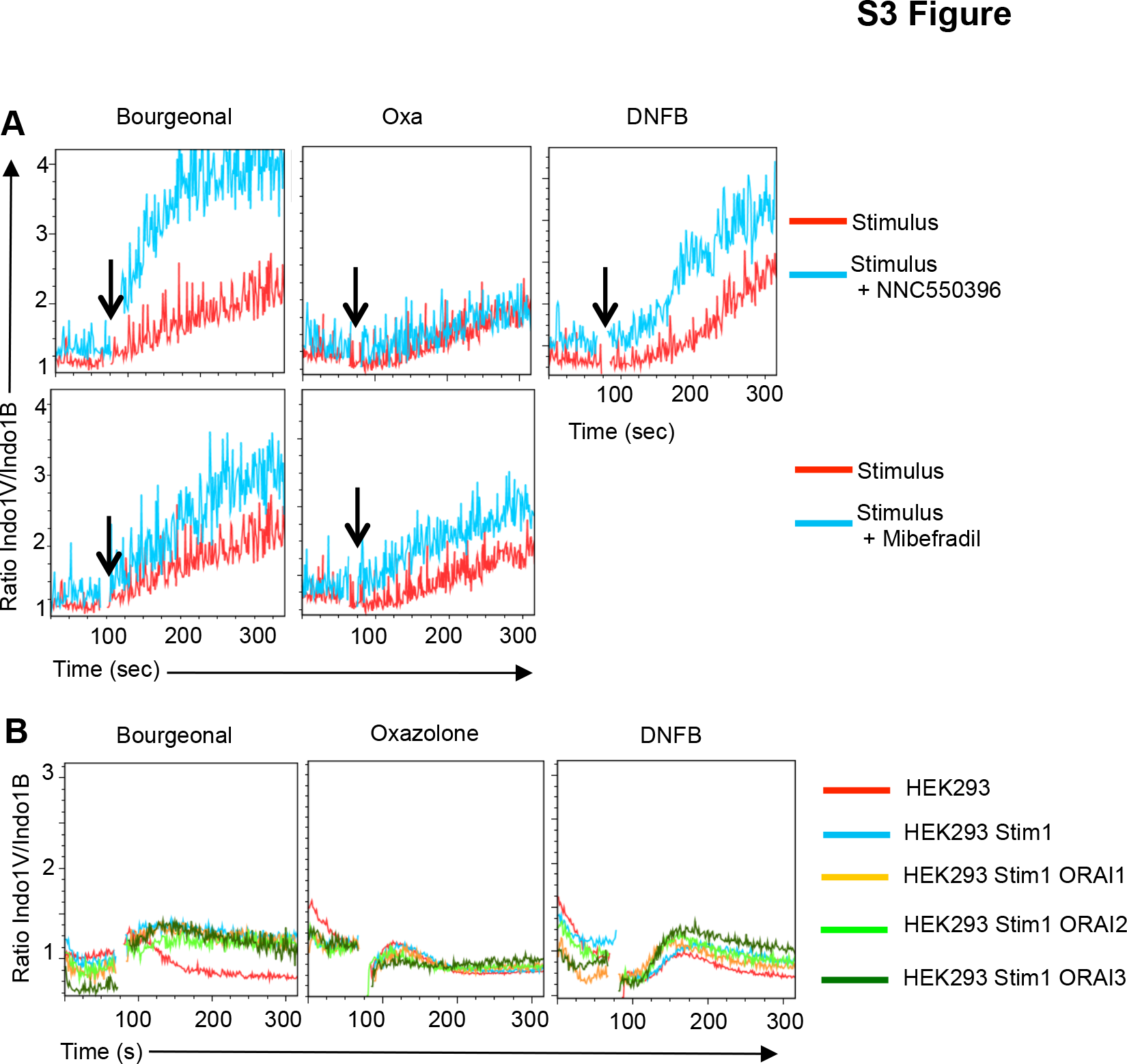

Supplement: S3 Fig — A) CatSper inhibitors do not block hapten-induced Ca2+ entry into NK cells. The CatSper inhibitors NNC55-0396 (1 μM) and Mibefradil (5 μM) do not to reduce Bourgeonal, Oxa or DNFB-induced Ca2+ entry into primary NK cells. Rather these compounds may enhance Ca2+ entry. B) HEK293 cells were stably transfected with hSTIM1 without or with hORAI1, hORAI2 or hORAI3. Bourgeonal (100 μM) Oxa (0.4 mM) and DNFB (0.25 mM) failed to induce Ca2+ flux in any of the transfectants. (TIF) [file pone.0151031.s003.tif]

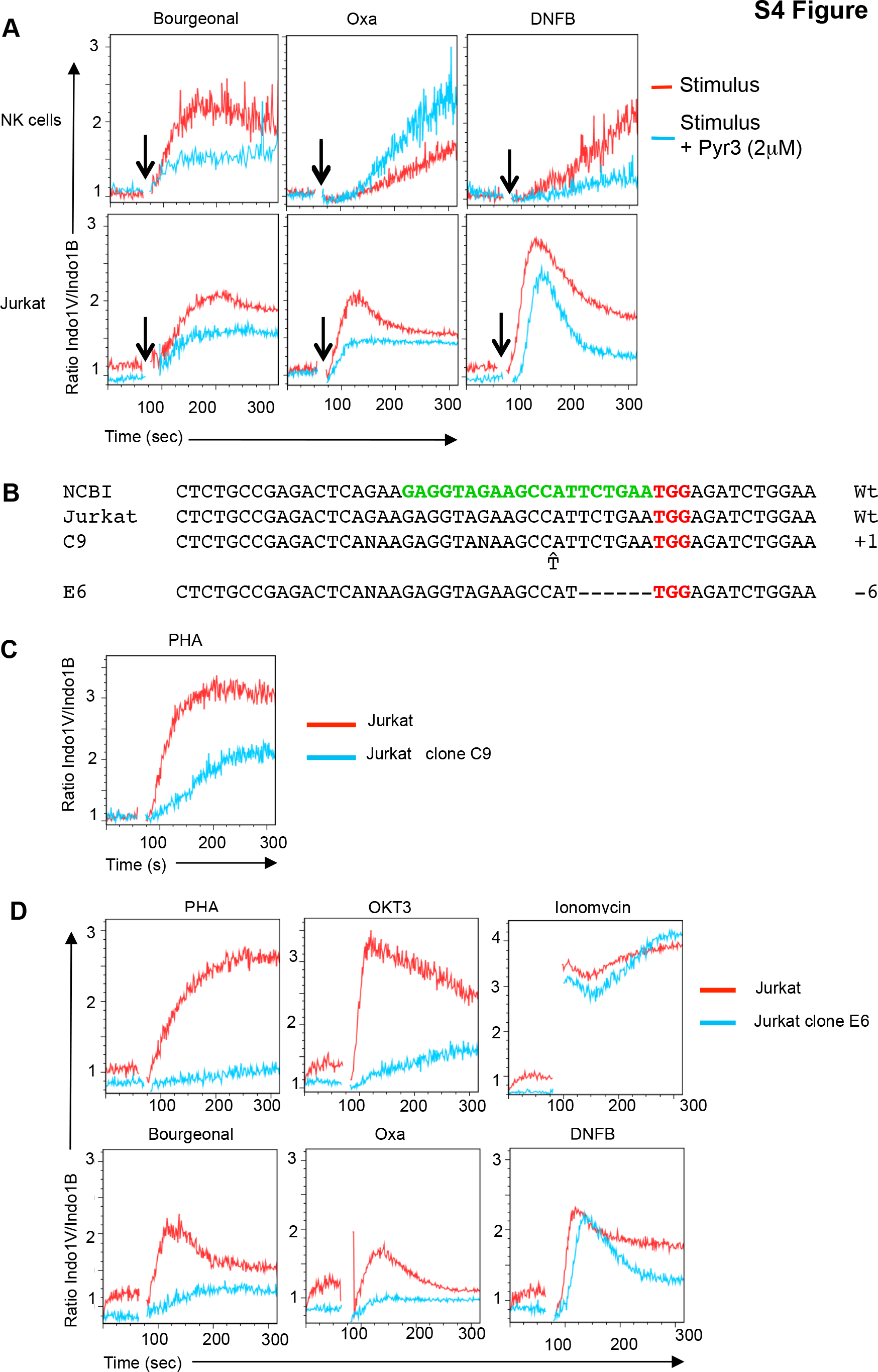

Supplement: S4 Fig — A) Ca2+ entry into gated NK cells (top) or Jurkat cells (bottom) induced by Bourgeonal (100 μM), Oxa (400 μM) or DNFB (500 μM for NK cells and 100 μM for Jurkat cells) in the presence of a low dose of Pyr3 (2 μM). B). Sequence of the targeted portion of TRPC3 available in NCBI, determined in wild type Jurkat cells and in the mutant clones C9 and E6. The TRPC3 species amplified from C9 has a 1bp insertion (+1), which leads to a premature stop and thus loss of function. E6 has a 6bp deletion, which removes 2 amino acids from the cytoplasmic portion of TRPC3. E6 is thus likely a hypomorphic rather than a null mutation. The sgRNA-targeting the TRPC3 sequence is shown in green, the protospacer-adjacent motif (PAM) sequence is in red. C) Ca2+ flux response induced by Phytohaemagglutinin (PHA) (50 μg/mL) in Jurkat cells (red line) and TRPC3 mutant Jurkat clone C9. D) Ca2+ entry into TRPC3 mutant clone E6 (blue line) and wild type Jurkat cells (red line) induced by Phytohaemagglutinin (PHA) (50 μg/mL), OKT3 antibody (2 μg/mL), Ionomycin (1 μg/mL), Bourgeonal (100 μM), Oxa (0.4 mM) or DNFB (0.25 mM). (TIF) [file pone.0151031.s004.tif]

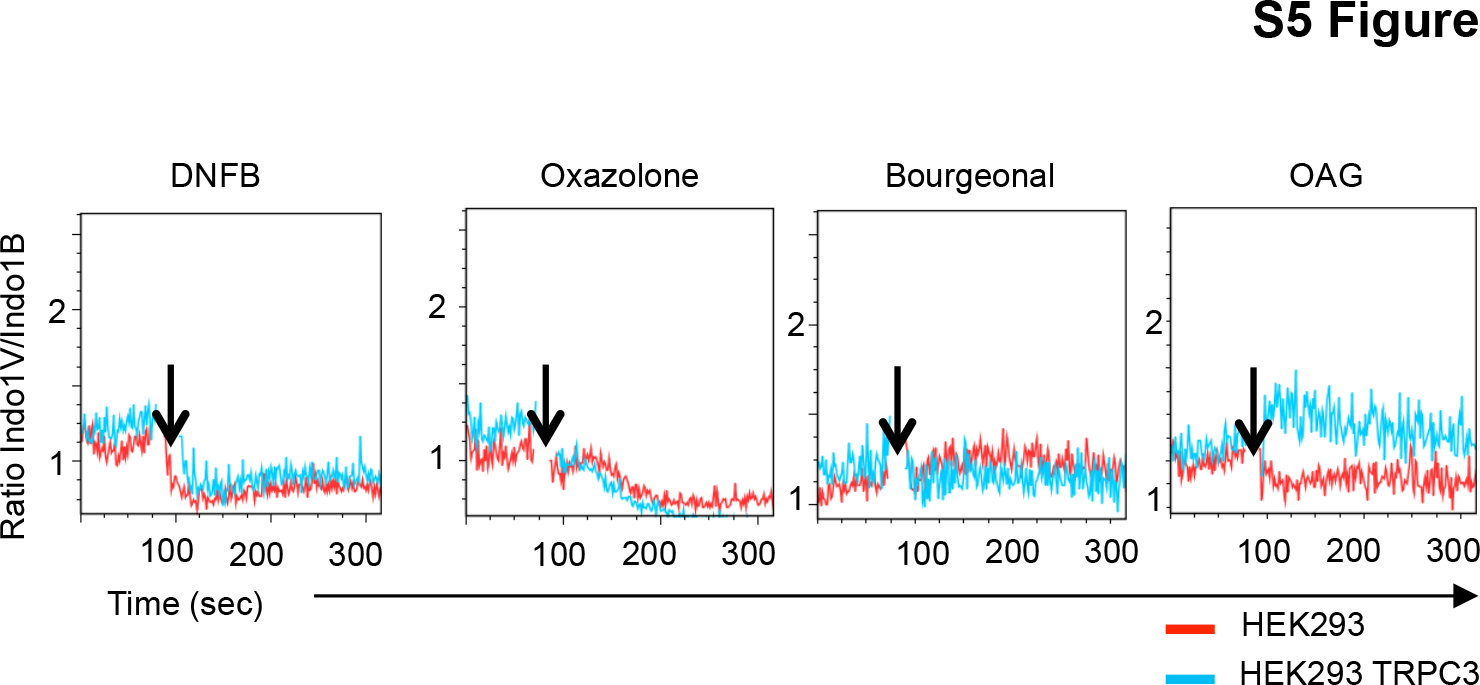

Supplement: S5 Fig — HEK293T were stably transfected with TRPC3 cDNA (blue line) and stimulated with Bourgeonal, Oxa or DNFB or the TRPC3 ligand 1-oleoyl-2-acetyl-sn-glycerol (OAG). (TIF) [file pone.0151031.s005.tif]
