# Supplementary material for: NK Cells Respond to Haptens by the Activation of Calcium Permeable Plasma Membrane Channels
Source: PLoS One. 2016 Mar 10;11(3):e0151031. doi: 10.1371/journal.pone.0151031 (PMC4786276; doi:10.1371/journal.pone.0151031)
Supplement: S1 Table — Analysis of bone marrow NK cells from wild type mice for the expression of OR and selected G-proteins genes in bone marrow NK cells. Shown are the 20 most highly expressed OR genes and selected G- Proteins. (DOCX) [file pone.0151031.s006.docx]

**S1 Table: Expression of genes coding for OR and G-proteins**

**in NK cells**

| ID | WT mean | Gene Accession | Gene Symbol |
| --- | --- | --- | --- |
|  | (log 2) |  |  |
|  |  |  |  |
| 10598105 | 6.59 | NM_207175 | Olfr239 |
| 10376412 | 6.35 | NM_001011760 | Olfr314 |
| 10375527 | 6.04 | NM_146276 | Olfr1394 |
| 10450872 | 6.02 | NM_146515 | Olfr99 |
|  |  |  |  |
| 10484805 | 5.55 | NM_001011761 | Olfr1229 |
| 10389670 | 5.14 | NM_146413 | Olfr463 |
| 10556033 | 5.03 | NM_001005570 | Olfr707 |
| 10559784 | 5.01 | NM_146385 | Olfr1347 |
| 10426592 | 5.25 | NM_146457 | Olfr282 |
| 10389668 | 5.18 | NM_146411 | Olfr462 |
| 10572743 | 5.11 | NM_146539 | Olfr373 |
| 10512626 | 5.56 | NM_019486 | Olfr71 |
| 10566140 | 5.72 | NM_146840 | Olfr545 |
| 10375797 | 5.15 | NM_010997 | Olfr54 |
| 10388308 | 5.38 | NM_146709 | Olfr411 |
| 10568954 | 5.44 | NM_001011814 | Olfr524 |
| 10375525 | 5.15 | NM_146877 | Olfr1395 |
| 10450854 | 5.82 | NM_182714 | Olfr91 |
| 10555907 | 5.20 | NM_146758 | Olfr678 |
| 10378427 | 5.82 | NM_146706 | Olfr401 |
| 10348641 | 5.57 | NM_146491 | Olfr1410 |
|  |  |  |  |
| 10456363 | 3.55 | NM_010307 | Gnal |
| 10461844 | 6.23 | NM_008139 | Gnaq |
| 10371230 | 7.73 | NM_010301 | Gna11 |
